# Transforming P & O Care with 3D Printing–More than Meets the Eye

**DOI:** 10.33137/cpoj.v6i2.42138

**Published:** 2023-12-22

**Authors:** J Andrysek, S Ramdial

**Affiliations:** 1 Bloorview Research Institute, Holland Bloorview Kids Rehabilitation Hospital, Toronto, Canada.; 2 Institute of Biomedical Engineering, Faculty of Applied Science and Engineering, University of Toronto, Toronto, Canada.; 3 Orthotics and Prosthetics Department, Holland Bloorview Kids Rehabilitation Hospital, Toronto, Canada.

**Keywords:** Prosthetics, Orthotics, Fabrication, Additive Manufacturing, 3D Printing, Digital Workflows, Scanning, Socket

## Abstract

Many within the prosthetics and orthotics (P&O) industry are embracing 3D printing technology to produce better devices more efficiently, cost-effectively and to improve patient outcomes. 3D printing is here to stay, but how much will it transform P&O practices? This paper explores the state-of 3D printing technology as it applies to P&O and aims to highlight important considerations for bringing 3D printing into mainstream practice. The paper draws from recent published literature, as well as experiences stemming from ongoing efforts focused on implementing digital workflows and 3D printing into P&O care. The paper examines the topic from the technological, research, economics, funding, and clinical perspectives. While 3D printing and digital workflows have advantages over traditional methods (i.e. ability to design more complex parts, reprinting and reproduction of parts, less labour intensive) there are also challenges limiting adoption. First, despite recent advancements in 3D printing technology, gaps still exist in terms of the materials and processes. For example, cost-effectively fabricating devices that are concurrently strong and durable, allow for colourful designs, and are thermoformable are still being developed. Cost-wise, 3D printing may currently be more viable for small, or paediatric devices. There are also limited technical standards to ensure safe and durable devices are produced, as well as a lack of evidence and information about patient outcomes and operating costs. Nevertheless, a great amount of enthusiasm and momentum exists within the industry to innovate, and with it the potential for 3D printing to one day be central to mainstream P&O care. Given the many aspects of the P&O industry, collaboration and partnerships will facilitate learning from each other to advance and realize the potential of 3D printing sooner.

## INTRODUCTION

With custom care being a prominent focus in the Prosthetics and Orthotics (P&O) industry, the ability to create unique assistive devices suggests 3D printing is the ideal solution. The appeal of additive manufacturing is its ability to produce one-off parts relatively quickly and inexpensively. While 3D printing has been around for decades, recent technological advancements have made it more relevant, meeting the design and functional requirements of the P&O field. But is 3D printing poised to transform P&O practices, and replace our traditional methods? This paper provides insight on this by examining the state-of 3D printing technology as it applies to P&O, and highlighting important considerations in bringing 3D printing into mainstream practice. The paper draws from recent published literature, as well as our learnings of 4+ years of work to implement digital workflows and 3D printing into the clinical services within our own rehab hospital. The intention of this paper is to provide useful information and guidance to the P&O sector including practitioners (i.e. clinicians and technicians implementing 3D printing in their practices), manufacturers (those developing digital workflows and 3D printers), researchers, health care funders and policy makers, users, educators, and the media, which has not always presented the topic in an accurate way.

## 3D PRINTING TECHNOLOGY

3D printing allows for the construction of three-dimensional objects from a digital model by joining or solidifying materials. Many different 3D printing methods exist, utilizing a variety of materials. Common printing methods include fused deposition modeling (FDM), whereby an object is built-up with an extruded filament, sintering, where a powder is fused together, or stereolithography, in which liquid resin is hardened into the desired object. Polymers (plastics such as PVA, PLA, PET, ABS, nylon) are the most commonly printed materials and relevant to P&O, but metals, resins, and even composites can also be 3D printed.

Each printing method is unique in terms of the materials that can be used, the characteristics of the fabricated parts (i.e. part geometries and size, surface finish, colors, strength, cost), and general usability (i.e. cost of or access to printer, printing time, and post processing requirements). It is crucial to highlight that no single printing technology can do it all, and the most likely scenario is that clinics will need access to a multitude of different printers, depending on their applications. For example, implementation efforts may rely on the use of a low-cost FDM printer for fabricating check or diagnostic sockets while a sintering printer such as the Hewlett Packard (HP) Multi Jet Fusion (MJF) is used for the final prosthesis or orthosis requiring high strength and durability.

## 3D PRINTING IN P&O

Before diving further into 3D printing in P&O, it is important to recognize that 3D printing is closely integrated with digital workflows (i.e. the process of developing the digital models that are ultimately printed). Typically, this includes some method of scanning the user's body part and using Computer-Aided Design (CAD) software to develop the digital model. For decades, these digital workflows have included carving at the latter end to make a foam positive for molding a brace or socket, however, 3D printing has the potential to replace this final step. Within our own work, it has taken considerable time and effort to figure out the digital processes for different devices (determining what scanner and design software are best, learning how to effectively use them, establishing file management etc.). Applying digital workflows into P&O practice has its challenges and benefits. Challenges include: incomplete solutions, inability to work with physical models, and the costs and investment needed to implement them, among others.^1^ However, these can be viewed as short-term hindrances as there are also benefits — the first of which being the possibility of more efficient processes for making and remaking devices. Once a digital model is developed, it can be easily adjusted (digitally) and/or printed as many times as needed. If a user breaks their device, another one can be reprinted and sent to them. Within our work, users have expressed their desire to have fabrication processes that require fewer visits to the clinic or hospital. Another benefit is the opportunity for large data sets of digital models to inform best clinical practices around the design of devices, and to advance the design processes to be more scientifically driven.^1^ This could help in teaching and training, or to automate aspects of the design process to improve usability and efficiency of CAD programs; ultimately improving outcomes by allowing devices to be fabricated more consistently.

Beyond developing and implementing workflows for making sockets and orthoses, our group has also explored the use of 3D printing in a number of other ways. The first among these is the design of terminal devices that do not exist commercially, including a custom hand,^2^ as well as recreational devices (such as a hockey stick attachment for a youth with a transradial prosthesis). We have also used 3D printing in the design and development of commercial prosthetic components such as the All-Terrain prosthetic knee joint. The versatility of 3D printing makes it possible to explore applications that were not previously possible. For example, a market has evolved around 3D printed personalized cosmetic prosthetic covers, enabling disability to be fashionable rather than something that people try to conceal. Similarly, greater personalization and customization of prosthetic liners is now possible with the advent of 3D printing of silicone. A liner can be printed with varying materials and thicknesses, to better conform and transfer the loads to the limb to improve comfort and function. Variable thickness also enables orthotic and prosthetic interfaces to be designed to have compliance in certain areas and provide greater support in others, to improve comfort and load bearing capabilities.

As previously mentioned, implementing digital workflows and 3D printing in P&O practice presents some unique challenges, and many are related to 3D printing technology itself. Until recently and before the introduction of 3D printing technology such as the HP MJF, cost-effectively printing larger parts that met their strength requirements was a major limitation.^3^ However, the capital investments of acquiring a MJF printer are substantial, as are operational costs which can include such aspects as hiring a trained technician to run the equipment. At present, there is limited information on the economic aspects of using digital workflows and 3D printing as compared to traditional workflows, making it a leap of faith for those interested in implementing these new technologies.^4^ An additional financial consideration which remains a gray area in some health care systems, is reimbursement. However, professionals and the P&O industry are advocating for approval of new and proven technologies.

Beyond the economic aspects, concerns also persist about the suitability of existing 3D printing technology for use in P&O. An important aspect is strength and durability, given the critical role that P&O devices play in the lives of their users. Strength is determined not only by the material used, but also the printing process. For example, FDM printed parts are prone to weakness between printed layers, resulting in potential fracture points. Sintered parts (such as with the MJF) are much more isotropic and avoid this but come at a much higher cost as previously mentioned. A related challenge is the absence of standards or protocols for strength testing components such as sockets and orthoses. The design of custom P&O devices such as sockets or orthoses is largely based on best practices and the practitioner's experience, and such experiential evidence is still limited for non-traditionally made devices. Structural testing standards such as ISO 10328 do not cover P&O components beyond prosthetic knee joints and feet.

Further, the diversity of printing materials and methods, as well as device requirements, complicates the establishment of accepted standards and practices. Nevertheless, general guiding principles for 3D printing medical devices are being established. The standards identify important technical considerations for manufacturing and testing of 3D printed medical devices including point-of-care and patient-matched (custom) devices which are relevant to the P&O industry.^5^

Other characteristics are also important, but not easy to achieve with existing 3D printers. Printed materials should ideally be thermoformable to allow for both immediate adjustments to parts such as sockets and orthoses during fitting and future adjustments due to growth or other physiological changes. However, the most promising printing methods (such as the HP MJF) are not ideal in this respect. The sintered MJF Nylon material has limited thermoformability, and the application of heat has the unfortunate effect of changing the color and finish of the surface, thus compromising aesthetics of the finished piece as well as possibly the mechanical properties. The MJF printer is also limited in terms of colors that can be printed, with dark grey being the default. HP previously sold a printer that gave us a variety of choices, but these colour features are no longer available. This is unfortunate, as many users (kids and adults alike) see color as an important aspect of device personalization. In contrast, FDM printers can easily print in color, however, as noted earlier the strength and surface finish are questionable. In the case of diagnostic sockets that need to be transparent in addition to being thermoformable, the options are even more limited. Curable resins that are transparent are not thermoformable. Plastics used in FDM are thermoformable, but the FDM process limits transparency due the way the filament is layered to make the parts. Optimization of printing parameters and surface treatments can help to increase transparency, but currently there is little guidance on how to achieve acceptable results in practice. Trade-offs exist, and many of these unique challenges are left up to the P&O industry to find solutions.

Unraveling more of the details, there are other challenges to overcome. For example, currently there is no simple way to digitally capture the changes made by heat forming a diagnostic socket for the finalization of the model and printing of the definitive socket. Scanners that can effectively capture the inside shape of a socket, especially small sockets, are not available. We even went as far as trying dental scanners, but these are not designed to reconstruct topologies relevant to P&O devices. One could fill the diagnostic socket with plaster or alginate and then digitally scan in this positive model, but it would be ideal if this “traditional workflow” step could be avoided. Another restriction is size. Not only does the cost of the printed part increase with size, but most printers have print volumes that cannot accommodate larger devices. For example, current MJF printers have a build volume that can print a smaller AFO, but not a large adult one. Joining two printed parts to make a larger device is possible but requires extra steps and may compromise strength. 3D printing at present may be best suited for smaller devices such as foot orthotics, and also pediatric over adult care.

The possibilities that 3D printing offer are exciting. One only needs to go to any P&O forum or congress, to see all of the innovation that companies, researchers, and health care are driving. From improvements to digital design and data management software and new printing technologies, to service bureau models for central fabrication and beyond. However, current 3D printing technology does not fully meet the needs of the P&O industry and as such, the future remains uncertain. The ideal 3D printer would be cost-effective and not only produce strong and durable parts with a good finish, but also colors and designs that users desire. Processes and materials that are concurrently able to provide the important features (strength, durability, good finish, thermoformability, colors, biocompatibility etc.) are needed. Additionally, reduced print times (i.e. < 1 hour) would allow devices to be fabricated and tested during a single session, thus reducing the number of appointments and overall healthcare costs and burden on users. It is a lot to ask for and expect from 3D printing to meet all these criteria. The alternative is that clinical practices continue to adapt to best utilize the existing tools. The mainstream adoption of 3D printing in P&O would change practice, and it must not compromise quality of devices, treatment and services. Given the diversity of P&O care, it will continue to take ingenuity and craftiness to address the many practice nuances over time as was done with traditional processes. One important aspect of this, is that we are able to learn from each other. In our efforts to implement digital technology workflows, we have been very fortunate at the willingness of other clinics, hospitals, researchers and even companies to share their expertise and learnings with us. These partnerships help us move forward with greater ease and to apply digital workflows in our care. It is our goal to reciprocate and support others. We need to strive to continue to bring the communities interested in digital workflows and 3D printing together. Some considerations include establishing special interest groups, forums or networks and to collaborate globally (i.e. International Society for Prosthetics and Orthotics - ISPO). The discussions must also involve manufacturers, who can ultimately help address the limitations of existing printing technology, and policymakers and funders, who can help to remove funding and regulatory barriers in how we can implement 3D printing in practice.

## CALL TO ACTION

Is 3D printing the future of P&O care? Will it continue to be a way that we make the occasional one-off device, or will it more profoundly transform our practice? It is too early to know and will depend on the future advancements in the technology itself, the P&O ecosystem, the validation from all involved — especially our users, and also our continued willingness and ability to work toward implementing it. The driving elements, and metrics to indicate that we are on the right track, will boil down to whether the needs of clients can be served more efficiently and effectively. This will require shared learnings from the users, clinical, technical and research groups, and utilizing available evidence about the quality of care and cost-effectiveness in comparison to the status quo.

## DECLARATION OF CONFLICTING INTERESTS

The authors have no conflicts of interest to declare related to this paper.

## AUTHORs CONTRIBUTION

Both authors contributed equally to the research and the writing of this manuscript.

## SOURCES OF SUPPORT

Holland Bloorview Foundation Grants from 2021 to 2023. Natural Sciences and Engineering Council (NSERC) Discovery Grant 493032. NSERC Alliance Grant 514883. The War Amps.

## AUTHORS SCIENTIFIC BIOGRAPHY

**Figure:**
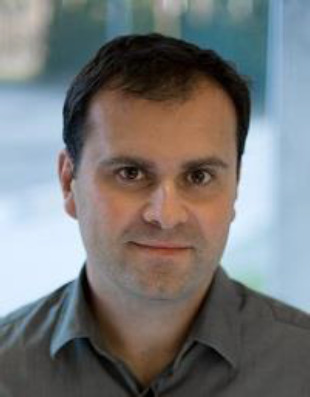


**Dr. Jan Andrysek** is a Senior Scientist at the Bloorview Research Institute of Holland Bloorview Kids Rehabilitation hospital and an Associate Professor at the Institute of Biomedical Engineering, University of Toronto. His research program focuses on the development of treatments and assistive technologies for children and youth with physical disabilities. Specific areas of study include prosthetic and orthotic limb design and control, bio sensing, biofeedback and gait training systems, and understanding the global need for prosthetic and orthotic technology and its impact on mobility, physical function, and quality of life. He is the recipient of awards including the 2017 Ontario Profession Engineers Engineering Medal for Research and Development. In 2019 Dr. Andrysek was elected an American Institute for Medical and Biological Engineering (AIMBE) fellow. He is also the co-founder and Chief Scientific Officer at Legworks Inc., a social for-profit enterprise focused on improving prosthetic technologies and care for individuals globally.

**Figure:**
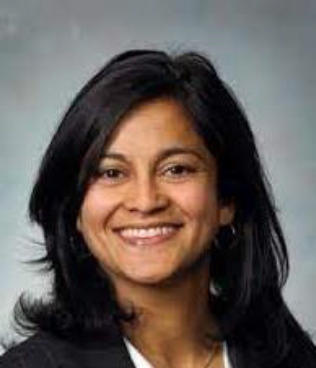


**Sandra Ramdial** is a certified prosthetist. She is the past operations manager for the Orthotic and Prosthetic Department at Holland Bloorview Kids Rehabilitation Hospital where she led a group of clinical and technical professionals and had direct involvement in client care. She has over 35 years of experience in the field including 22 years at Holland Bloorview and was part of the Professional and Clinical Services team at Otto Bock Healthcare Canada where she managed the Custom Silicone Group and a key clinical ambassador and educator for upper limb prosthetics. Sandra is the past president of the Canadian Association of Prosthetics & Orthotics and the International Society for Prosthetics & Orthotics (ISPO) Canada and the past secretary-treasurer for the Association of Children's Prosthetic-Orthotic Clinics. She continues to be involved in Orthotics & Prosthetics and is presently the President Elect for the International Board of ISPO. In addition to her extensive experience, she brings an even greater amount of enthusiasm and passion for research and development, and new technologies to the field.

## References

[1] Ngan C, Sivasambu H, Kelland K, Ramdial S, Andrysek J. Understanding the adoption of digital workflows in orthotic & prosthetic practice from practitioner perspectives: a qualitative descriptive study. Prosthet Orthot Int. 2022;46(3):282–289. DOI: 10.1097/PXR.0000000000000107 35315819

[2] Eshraghi A, Yoo J, Klein J, Mckenzie I, Sebaldt G, Leineweber M, et al. A custom, functional and lifelike passive prosthetic hand for infants and small toddlers: clinical note. Prosthet Orthot Int. 2020;44(3):180–184. DOI: 10.1177/0309364620909276 32301382

[3] Ribeiro D, Cimino SR, Mayo AL, Ratto M, Hitzig SL. 3D printing and amputation: a scoping review. Disabil Rehabil Assist Technol. 2021;16(2):221–240. DOI: 10.1080/17483107.2019.1646825 31418306

[4] Roberts A, Wales J, Smith H, Sampson CJ, Jones P, James M. A randomised controlled trial of laser scanning and casting for the construction of ankle-foot orthoses. Prosthet Orthot Int. 2016;40(2):253–61. DOI: 10.1177/0309364614550263 25336052

[5] Technical considerations for additive manufactured medical devices: Guidance for Industry and Food and Drug Administration Staff [Internet]. Food and Drug Administration. 2017. [cited Sep 22, 2023]. Available from: https://www.fda.gov/regulatory-information/search-fda-guidance-documents/technical-considerations-additive-manufactured-medical-devices

